# Evidence for Diverse Prognosis in High-Grade Serous Ovarian Carcinoma: Solid, Pseudoendometrioid, and Transitional-Like; So-Called “SET Morphology” and Progesterone Receptor Status

**DOI:** 10.5146/tjpath.2022.01571

**Published:** 2022-09-15

**Authors:** Halit Uner, Metin Demir, Dincer Goksuluk, Ayse Kars, Meral Uner, Alp Usubutun

**Affiliations:** Department of Pathology, Hacettepe University School of Medicine, Ankara, Turkey; Department of Medical Oncology, Hacettepe University School of Medicine, Ankara, Turkey; Department of Biostatistics, Hacettepe University School of Medicine, Ankara, Turkey

**Keywords:** Epithelial ovarian cancer, Prognosis, Morphology, BRCA1, Immunohistochemistry

## Abstract

***Objective:*** High-grade serous ovarian carcinoma (HGSC) is one of the major tumors of the gynecological system with a poor survival rate and variable microscopic appearance. It was suggested that SET (solid, pseudo-endometrioid and transitional-like) morphology in ovarian HGSC is predictably associated with BRCA deficiencies. In this study, we investigated the microscopic patterns and some immunohistochemical markers predicting the prognosis of serous carcinoma.

***Material and Method:*** We re-evaluated 305 HGSC ovarian resections morphologically and calculated the SET morphology percentages for each case. Morphological and immunohistochemical data correlated with the survival and post-treatment disease progression data.

***Results:*** The median age at diagnosis was 57 years and the median follow-up period was 3.1 years. The median overall survival (OS) of ovarian carcinoma in SET-predominant tumors (n=60) was 81 months, while for tumors with SET non-dominant morphology (n=63) and non-SET morphology (n=182) it was 59.7 and 44.7 months, respectively.

***Conclusion:*** Predominant (more than 50%) SET morphology was significantly associated with increased survival rates of HGSC. Immunohistochemically, p53, ERCC1, ER, and PR antibodies were applied and only PR antibody positivity was found to be associated with borderline statistical significance for increased survival rates. Our results suggest that SET morphology may be a potential predictive and prognostic marker in managing the treatment strategies of HGSC.

## INTRODUCTION

Ovarian cancer is associated with a high mortality rate among women (1). More than 70% of ovarian cancers are diagnosed at an advanced stage and the absence of an accepted early diagnostic method is one of the main reasons for the increased mortality. The World Health Organization (WHO 2020) has classified epithelial ovarian tumors according to the cell of origin, and serous carcinomas have been separated into low- and high-grade categories (2). Approximately 90% of ovarian cancers are epithelial in origin and serous carcinomas comprise the majority (3).

Previous theories have suggested that ovarian cancers arise from ovarian surface epithelium derivatives like inclusion cysts, etc. (4,5). However, advanced sampling methods of fallopian tubes in prophylactic salpingo-oophorectomy materials obtained from *BRCA* mutated hereditary ovarian cancer syndrome patients have radically changed our traditional beliefs (6). Early intraepithelial carcinomas are reported at a fallopian tube location in BRCA deficient patients and currently, it is widely accepted that the majority of high-grade serous carcinomas (HGSCs) are tubal in origin. Approximately 10% of HGSC develop via *BRCA*1/*BRCA*2 mutations and the lifetime risk of ovarian cancer in *BRCA* mutated patients is around 50% (7). Recently Howitt et al. have reported that the coincidence of intraepithelial lesions (serous tubal intraepithelial carcinoma - STIC) is relatively rare in *BRCA* mutated serous carcinomas (30%), while sporadic HGSCs may contain up to 60% of tubal precursor lesions (8,9). The difference between these tumors is not only limited to the presence of precursor lesions but there are also various morphologic features concerned. Soslow et al. have reported that the morphologic appearance of HGSC in patients with BRCA abnormality differs from the morphologic appearance of those without this abnormality and called the morphologic appearance of *BRCA* mutated patients as SET (10). This morphology consisted of appearances as “Solid”, “pseudo-Endometrioid”, and “Transitional cell carcinoma-like” patterns. They found that these morphologic patterns are more common in patients with BRCA abnormalities, and suggested that this indicates a potential relationship between morphology and genotype.

There are few prognostic studies in *BRCA* mutated patients with ovarian serous carcinoma, and two recent studies suggest that these patients may have better survival (11,12). Theoretically, the *BRCA* mutated group is more sensitive to DNA crosslinking agents such as platinum-based chemotherapeutics due to a lack of DNA repair mechanism of the *BRCA* gene. However, eventually almost all high-grade serous carcinomas develop resistance to platinum (13). Increased metabolism of platinum and efflux of platinum or increased repair of platinum-induced DNA cross-links are possible mechanisms of resistance. Repair of platinum-induced DNA cross-links could be possible with excision of nucleotides primarily by excision repair cross-complementation 1 (ERCC1) and other nucleotide excision proteins.

The objective of this study was to investigate the effect of SET morphology on clinical outcome and expression of certain proteins such as p53, ER, PR, and ERCC1 in patients with tubo-ovarian high-grade serous carcinoma.

## MATERIALS and METHODS

### Case Selection

The study was approved by the Hacettepe University non-interventional Clinical Research Ethics Committee (approval number: GO 15/454-26). The pathology reports of patients diagnosed as “serous carcinoma of the ovary” between 2001 and 2015 were searched from the hospital information system of Hacettepe University Medical School. Four hundred sixty-seven patients were found with this diagnosis in our pathology files. Seventy-one cases were consultation cases and we did not have their paraffin blocks. There were 68 recurrent cases where the primary operation was not performed in our institution. Both patient cohorts were excluded, respectively, due to inability of additional laboratory studies and possible morphologic alterations following treatment. The slides of the remaining cases were retrieved from the archive and re-examined by two pathologists (AU, HU). After reviewing the selected cases according to the WHO 2020 criteria, 23 samples were found to have different morphologies than HGSC such as low-grade serous carcinoma, borderline serous tumors, etc. After excluding these cases, 305 ‘high-grade serous ovarian carcinoma’ cases were included in the study.

### Microscopic Evaluation

**Figure 1:**
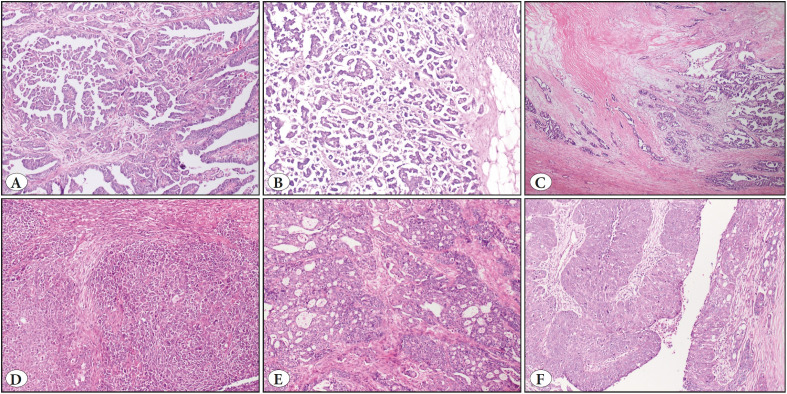
Architectural patterns of high-grade serous carcinoma (H&E, X40). **A)** Papillary pattern. **B)** Micropapillary pattern. **C)** Infiltrative pattern. **D)** Solid pattern. **E)** Pseudoendometrioid pattern. **F)** Transitional pattern.

By microscopic examination of all the slides of a given case, invasive tumor patterns were noted while blinded to the clinical outcome. The latest WHO bluebook practically describes the morphological patterns of HGSC as papillary, glandular, slit-like glandular, and cribriform. We determined the detailed patterns of high-grade serous carcinoma as follows. *Papillary*; neoplastic cells covering central fibrous cores, one of the most common conventional patterns. *Micropapillary*; composed of non-epithelial lined spaces filled with solid tumor islands and neoplastic cells usually devoid of fibrovascular cores. *Infiltrative*; haphazardly distributed solid masses or less condensed tumor cells forming slit-like spaces. Infiltrating tumor masses were usually concomitant with stromal desmoplasia. *Solid*; the presence of bulky tumor islands without a specific growth pattern. *Pseudoendometrioid*; tumor cells forming gland-like structures with usually tubular cells, and also includes round spaces reminiscent of cribriform architecture. *Transitional*; the presence of tumor cells forming insular or trabecular architecture resembling the multilayered epithelium of bladder (Figure 1). Morphologies like papillary, micropapillary, and infiltrative pattern were more commonly observed and included in the conventional pattern (14). Recently described solid, pseudo-endometrioid, and transitional architectures were noted and graded as follows; tumors having less than 5% SET pattern (non-SET), tumors with more than 5% but less than 50% SET morphology (SET non-dominant), and tumors with more than 50% SET morphology (SET-predominant). Besides, all fallopian tube sections were evaluated for serous tubal intraepithelial carcinoma or tumor infiltration and also for other disorders. Fallopian tube sampling at our institution has improved over the years. Before 2008, transverse sections from the isthmic portion were submitted. However, from 2008 onwards, the fimbrial portion of the fallopian tube was added to the sampling in accordance with the guidelines. Because of the retrospective nature of the study, and the variable fallopian tube sampling at our institution, almost none of the sampling has been performed with the sectioning and extensively examining the fimbria (SEE-FIM) protocol, unless there was strong evidence that a patient could have syndromic manifestations.

### Immunohistochemical Study

Two foci were marked on the tumor slides and 3 mm samples were removed from the formalin-fixed paraffin-embedded tissue blocks to construct tissue microarray (TMA) blocks. If SET morphology was encountered within the tumor, at least one focus was sampled from that particular area. Several 4-5 micrometer thick sections obtained from TMA blocks were stained with hematoxylin and eosin (H&E) and immunohistochemical staining was performed on the Leica BOND-MAX IHC/ISH automated immunostainer using the following commercial antibodies; ER (Biocare; EDTA 1/50; 6F11), PR (Biocare; EDTA 1/100; SP2), p53 (Biocare; EDTA 1/200; DO-7) and ERCC1 (Biocare; EDTA 1/100; 4F9).

### Evaluation of Immunohistochemical Results

We used the College of American Pathologists Guideline recommendations for ER and PR immunohistochemistry, and at least 1% positive tumor cell nuclei was accepted as the threshold for a positive assay (15). Completely negative staining with p53 or overexpression in more than 80% of tumor cell nuclei widely known as ‘all or nothing’ indicating aberrant expression was accepted as “mutant” (16). Other staining patterns were included in ‘wild-type’ for p53. The staining intensity for ERCC1 was graded on a scale from 0 to 3 (a higher number indicating higher intensity) and the percentage of positive tumor cell nuclei was calculated according to the study by Park et al. (17).

### Survival

Demographic, clinical, and survival parameters were recorded from the hospital registration system. Overall survival (OS) information was also checked from the National Death Notification System. The time between the last cycle of first-line chemotherapy and the first cycle of the second-line chemotherapy was defined as time to progression (TTP). The duration from the operation date to the starting date of the first non-platinum chemotherapy was accepted as time to first platinum-free chemotherapy (TTFPFC).

### Statistical Evaluation

Data analysis was performed using the IBM SPSS software, version 21. Numerical variables were summarized using the mean and standard deviation or median and quartiles depending on the normality of the underlying distribution. The normality assumption was assessed using graphical (Q-Q plot, histogram, etc.) and analytical methods (Shapiro-Wilk’s normality test). Categorical variables were summarized with frequencies and percentages. Demographic variables were compared between SET morphology groups using chi-squared tests (e.g., Pearson, Fisher exact, etc.) for categorical variables and independent samples hypothesis tests (e.g., Student’s t-test or Mann-Whitney U test) for numerical variables, based on the normality of data. The effect of ER, PR, ERCC1, and SET morphology on survival was investigated using the Kaplan-Meier method and log-rank test. Survival times were reported using median times along with 95% confidence intervals. Pairwise comparisons were evaluated using Bonferroni adjusted p-values, and indicated with superscript letters. Groups not sharing similar letters are expressed as pairwise significant. Univariate Cox regression was used to find a list of possible risk factors regarding mortality, and pathologic features identified with univariate analyses were further entered into the multivariate Cox regression model to determine independent predictors of survival adjusted for the remaining risk factors. All test results were evaluated using two-sided p-values at the 0.05 level of significance.

## RESULTS

### Clinicopathological Data

The median age at diagnosis was 57 years (range 30-91). The tumor presented as a bilateral ovarian mass in 255 cases (83%). In 182 cases (60%), conventional serous morphology was completely dominant (only minuscule or no SET morphology), so-called non-SET. In 123 cases (40%), varying amounts of SET morphology were recorded (more than 5% of the tumor). In about half of these cases (60 cases) the SET morphology was more than 50%, so-called SET-predominant. In the rest of the cases (63 cases), SET morphology was between 5% and 50%, which was called SET non-dominant (Table 1).

**Table 1:** Patient demographics, stage, immunohistochemistry results and survival period based on morphology.

	**Non-SET (n=182)**	**SET non-dominant (n=63)**	**SET-predominant (n=60)**	
Mean age	59.6	57.4	57.8	
TNM				
Stage I	16/182 (8.8%)	10/63 (15.9%)	7/60 (11.7%)	
Stage II	8/182 (4.4%)	1/63 (1.6%)	4/60 (6.7%)	
Stage III	151/182 (83%)	51/63 (80.9%)	47/60 (78.3%)	
Stage IV	7/182 (3.8%)	1/63 (1.6%)	2/60 (3.3%)	
IHC				
wild-type p53	2/182 (1.1%)	0/63	2/60 (3.3%)	
high ERCC1	94/182 (51.6%)	28/63 (44.4%)	30/60 (50%)	
ER positivity	158/182 (86.8%)	56/63 (88.9%)	52/60 (86.7%)	
PR positivity	34/182 (18.7%)	19/63 (30.2%)	7/60 (11.7%)	
STIC (tubal sampling)	5/88 (5.7%)	2/30 (6.7%)	4/32 (12.5%)	
STIC (fimbrial sampling)	18/94 (19.1%)	7/33 (21.2%)	6/28 (21.4%)	
Outcome (months)				p (Log-Rank)
OS	44.74a (39.8, 49.7)	59.73a,b (47.7, 71.7)	80.98b (45.5, 116.4)	0.005
TTP	16.85a (9.8, 23.9)	23.78a,b (3.2, 44.3)	48.59b (18.6, 78.6)	0.013
TTFPFC	27.53a (18.4, 36.7)	50.36a,b (21.1, 79.6)	77.70b (41.3, 114.1)	0.003

**SET**: Solid, endometrioid and transitional, **IHC**: Immunohistochemistry, **ERCC1**: Excision repair cross-complementation 1, **ER**: Estrogen receptor, **PR**: Progesterone receptor, **STIC**: Serous tubal intraepithelial carcinoma, **OS**: Overall survival, **TTP**: Time to progression, **TTFPFC**: Time to first platinum-free chemotherapy *Median survival times were provided with 95% confidence intervals within parentheses. Pairwise comparisons of SET morphology were evaluated using the log-rank test of each pair, and a Bonferroni adjustment was performed on the p-values– Groups sharing similar letters are not statistically significant.

Fallopian tubes were free of tumor in 144 cases (47%). The tubes were infiltrated by the tumor in 119 (39%) of the remaining 161 cases. Only 42 cases (14%) were positive for intraepithelial lesion (STIC) in the fallopian tubes, and 23 of them had non-SET morphology while 19 had SET morphology. Even though we found no statistical significance, the overall survival time of patients with tumor-free fallopian tubes was 4 months longer compared to cases with a precursor lesion or tumor in tubal tissues, 43 versus 39 months respectively.

Eighty-two percent of the patients (249/305) had stage III disease according to the 2014 FIGO Staging System at the time of diagnosis. The distribution of stages was similar in the SET and non-SET morphology groups. Patients whose tumors had SET morphology were 2 years younger at the time of diagnosis compared to non-SET HGSCs but this difference was also not statistically significant (Student’s t-test, p = 0.12).

### Immunohistochemistry

**Figure 2:**
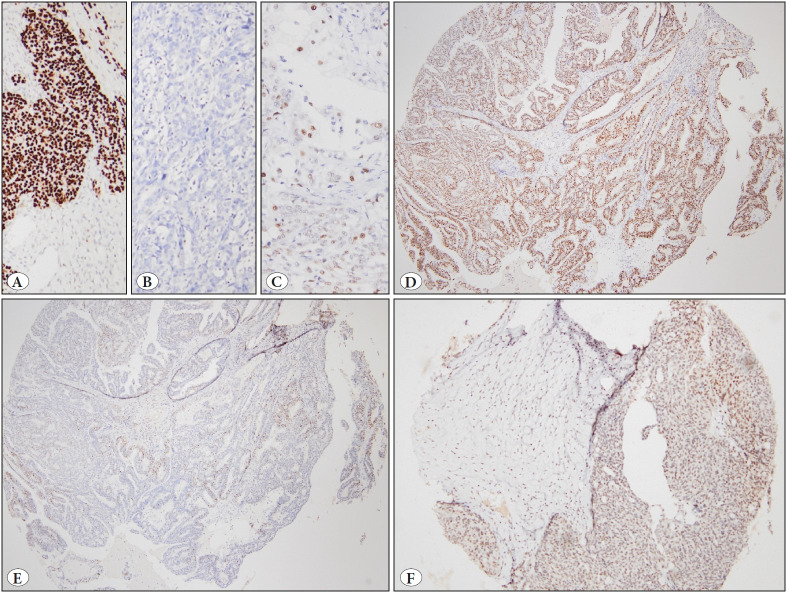
Immunohistochemistry. **A)** Diffuse strong nuclear staining with p53 (mutant). **B)** Total loss of expression for p53 (aberrant staining) that is also considered as mutant. **C)** Focal staining consistent with wild-type p53 expression. **D)** Higher ER positivity is usually seen in the diffuse pattern. **E)** PR expression is often seen as focal staining. **F)** High expression of ERCC1 (60% of cells stained as 2+).

The mutant type staining pattern of p53 was seen in 301 cases (99%) (Figure 2). Wild-type staining was observed in four cases only (Table 1, Figure 2). Two had SET-predominant morphology, while the other two had serous cancer with non-SET morphology. We included these cases in the study because of the presence of complex papillary structures, solid pattern, stratification of glandular cells, marked atypia, and high mitotic rates of conventional serous carcinoma.

Hormone receptor expression (ER and/or PR) was present in 269 cases (88%); 36 patients had no hormone receptor expression. ER expression was detected in 266 cases (87%) and PR expression in 60 (20%) cases (Figure 2). The majority of the tumors expressed ERCC1 (Figure 2), and only 15 cases (5%) out of 305 were ERCC1 negative. No significant correlation was found between the immunohistochemical markers and microscopic tumor patterns (Pearson’s chi-square, p = 0.79).

### Survival Data by Morphology

The median follow-up period was 3.1 years (range 0.1 - 12.5 years). One hundred fifty-nine patients had died, and 86 patients were still alive. The follow-up data of 245 cases were available, and that of the remaining 60 patients were missing. The ratio of patients who had progressive disease was 85% (n=259). Platinum resistance developed in 73% of patients. Platinum with or without taxane regimens were used in 81.6% of the patients as first line. The median number of chemotherapy lines administered was 2 (minimum: 0 - maximum: 11).

**Figure 3:**
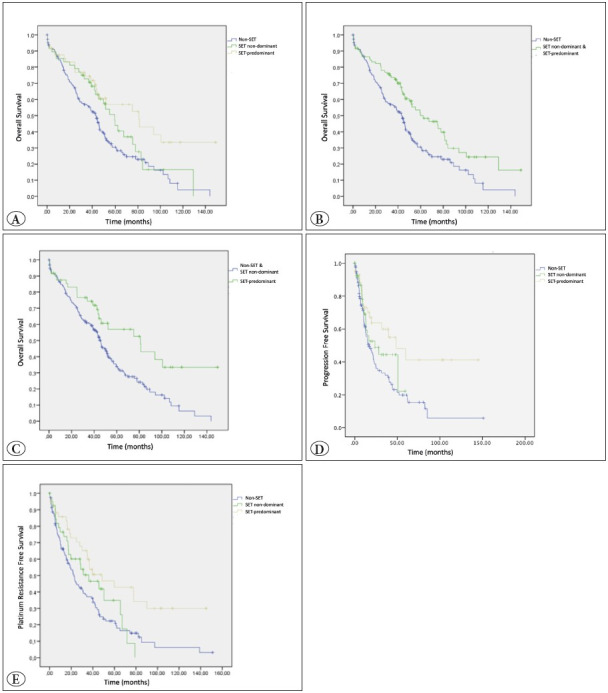
Survival analysis of the HGSC morphology groups; **A)** Overall survival rates of non-SET (blue), SET non-dominant (green), and SETpredominant (yellow) separately. **B)** Overall survival rates of non-SET (blue) versus SET non-dominant & SET-predominant (green) together. **C)** Overall survival rates of non-SET & SET non-dominant together (blue) versus SET-predominant (green). **D)** Time to progression (TTP). **E)** Time to first platinum-free chemotherapy (TTFPFC).

The median OS of high-grade serous carcinomas in non-SET (conventional) morphology and tumors with SET non-dominant morphology was 44.7 and 59.7 months, respectively, while the median OS of SET-predominant tumors was 81 months. The presence of SET morphology was a predictor of improved clinical outcome in OS analysis (p=0.005). Post-hoc analyses showed that the SET-predominant group had significantly higher median OS compared to conventional histology (p=0.004) (Figure 3, Table 1).

SET-predominant and non-dominant groups together had a significantly higher OS compared to the non-SET group (67.4 months vs. 44.7 months, p = 0.003) (Figure 3).

The SET-predominant group had a better outcome in terms of OS compared to SET non-dominant and non-SET tumors together, and this was statistically significant (81 months vs. 46.7 months, p = 0.006) (Figure 3).

In the entire cohort, the median overall survival (OS), median TTP and median TTFPFC were 50.4; 21.5, and 40 months, respectively. All these survival parameters showed only statistical significance between morphologic patterns. Median TTP for non-SET high-grade serous carcinomas, SET non-dominant, and SET-predominant groups were 16.9; 23.8, and 48.6 months, respectively (p=0.013). According to posthoc analyses, the SET-predominant group showed significantly higher median TTP compared to both the SET non-dominant (p=0.028) and non-SET groups (p=0.026) (Figure 3, Table 1). Median TTFPFC for non-SET high-grade serous carcinomas, SET non-dominant, and SET-predominant groups were 27.5; 50.4, and 77.7 months, respectively (p=0.003). The SET-predominant group had a higher median TTFPFC compared to the non-SET group with statistical significance (p=0.002) (Figure 3). The presence of SET morphology was a predictor of improved clinical outcomes for all survival parameters.

### Survival Data by Immunohistochemistry

The median OS of PR positive and negative cases were 66.8 months and 46.3 months respectively. This difference had a borderline statistical significance (p=0.059) (Table 2). ER expression, unlike PR, did not confer a survival advantage, and the overall survival in ER-positive and negative cases was 54 and 47 months, respectively.

**Table 2:** Univariate and multivariate Cox regression models of survival time.

**Time**	** **	**Simple Cox PH model**		**Multiple Cox PH model**	
	Variable*	**HR (95% CI)**	**p**	**HR**	**p**
Overall Survival	Morphology		**0.006**		**0.005**
	SET non-dominant		0.121	0.77 (0.51 – 1.17)	0.218
	SET pre-dominant		0.003	0.47 (0.3 – 0.75)	**0.001**
	ER (positive)	0.92 (0.56 – 1.49)	0.72	1.03 (0.62 – 1.70)	0.902
	PR (positive)	0.68 (0.452 – 1.02)	**0.059**	0.64 (0.42 – 0.97)	**0.034**
	ERCC1 (high)	1.01 (0.74 – 1.38)	0.966	1.07 (0.78 – 1.48)	0.663
Time to progression	Morphology		**0.015**		**0.017**
	SET non-dominant	0.73 (0.44 – 1.22)	0.227	0.81 (0.48 – 1.36)	0.424
	SET pre-dominant	0.48 (0.28 – 0.80)	**0.005**	0.47 (0.28 – 0.79)	**0.005**
	ER (positive)	0.98 (0.55 – 1.75)	0.951	1.04 (0.58 – 1.88)	0.885
	PR (positive)	0.84 (0.52 – 1.37)	0.488	0.81 (0.49 – 1.33)	0.41
	ERCC1 (high)	1.24 (0.85 – 1.80)	0.268	1.26 (0.85 – 1.86)	0.25
Time to first PFC	Morphology		**0.004**		**0.003**
	SET non-dominant	0.73 (0.44 – 1.21)	0.222	0.78 (0.47 – 1.30)	0.335
	SET pre-dominant	0.42 (0.25 – 0.70)	**0.001**	0.41 (0.24 – 0.69)	**0.001**
	ER (positive)	0.95 (0.53 – 1.69)	0.861	1.02 (0.27 – 1.85)	0.941
	PR (positive)	0.77 (0.48 – 1.26)	0.301	0.74 (0.45 – 1.22)	0.241
	ERCC1 (high)	1.13 (0.77 – 1.64)	0.535	1.22 (0.83 – 1.78)	0.319

**PFC**: Platinum-Free Chemotherapy, **ERCC1**: Excision repair cross-complementation 1, **PR**: Progesterone receptor, **ER**: Estrogen receptor * Non-SET is the reference category for morphology. Reference categories of the remaining categorical predictors were “negative” for ER and PR, and “low” for ERCC1

In 152 cases with high and 153 cases with negative and low ERCC1 expression, median OS was 49 months and 51 months, respectively. The difference was not statistically significant. Median TTP for the low and high ERCC groups was 35.6 (95% CI: 9.7-61.4) and 19.3 (95% CI: 14.8-23.8) months, respectively, and this difference was not statistically significant (p=0.27). Median TTFPFC for the same groups was 42.3 (95% CI: 34.8-49.7) and 34.4 (95% CI: 20.5-50.3) months, respectively (p=0.54).

In 4 cases who had wild-type staining p53, median OS was 47 months while in those with aberrant staining it was 41 months. There was no significant correlation between p53 staining and survival.

In the Cox regression analysis for OS, PR positivity was a positive factor for survival time (RR=0.64; 95% CI:0.42-0.96; p=0.031). SET predominance showed a decreased death risk compared to non-SET high grade serous carcinomas (RR=0.47; 95% CI:0.3-0.74, p=0.001). However, only SET predominance remained as the unique independent predictor of prevention from progression and platinum resistance compared to non-SET high grade serous carcinomas (RR for progression=0.47; 95% CI:0.28-0.79; p=0.004 and RR for platinum resistance=0.4; 95% CI:0.24-0.68; p=0.001) (Table 2).

## DISCUSSION

By reviewing a relatively large set of high-grade serous carcinomas, diagnosed according to WHO 2020 criteria, we demonstrated that these tumors could be potentially heterogeneous not only by their genetic and IHC profile but also by their morphological pattern and prognostic surveillance. In particular, increased interest in patients with high-risk hereditary cancers led to an increased understanding of the pathogenesis and morphological spectrum of serous cancers. In 2006, early tubal carcinomas were considered as precursors of high-grade serous carcinomas, and later in 2012, patients with *BRCA* mutations were found to have a different morphology such as SET (solid, pseudo-endometrioid, transitional carcinoma-like) compared to classical serous papillary carcinomas, and recently in 2015, lesser precursor lesions were reported in tumors with a *BRCA* mutation, leading to the opinion that serous carcinomas may reflect a more heterogeneous neoplastic development rather than a homogenous tumor group (6,9,10).

Based upon studies following risk-reducing salpingo-oophorectomy that detected serous carcinoma precursors in the fimbria of individuals with *BRCA* mutation, it was proposed that serous carcinoma originated in the fallopian tubes. These studies suggested that serous tubal intraepithelial carcinoma (STIC) was the prototype of serous carcinoma precursor. However, the detection rate of STIC in these individuals remained low. In early studies, STIC was reported in 10% of patients with *BRCA* mutation but in recent studies this rate goes up to 30% with a detailed sampling of the fimbrial end (9,18). On the other hand, the prevalence of STIC in sporadic high-grade serous carcinomas is around 60% (8). The majority of *BRCA* mutations are located in the *BRCA*1 gene and the *BRCA*2 gene mutations comprise fewer cases and the latter also demonstrates a smaller risk for early fallopian tube carcinomas (19).

HGSCs are the most common ovarian neoplasm in women with *BRCA* 1 and 2 mutations and there are numerous studies on this topic. *BRCA*1 and *BRCA*2 mutation rates reported as 10% of serous carcinomas earlier, increased up to 40% in recent publications (20,21). Contrary to BRCA, the incidence of SET morphology in serous carcinomas is not well known yet. In our study, 123 (40%) of 305 high-grade serous carcinoma cases showed SET morphology. These patients were 2 years younger than the patients with conventional morphology and, cases with SET were detected marginally more frequent at an early stage. The difference between the rates of STIC in SET and non-SET tumors was not notable. While cases with SET morphology in our study group were consistent with literature for age at presentation, the rate of STIC detection in these tumors was similar to that in conventional carcinomas. This difference might be due to fewer samples taken in the past at our institution during sampling procedures compared to the detailed SEE-FIM method and this may have caused very early precursor lesions to be missed in conventional cases.

The molecular detection of *BRCA* mutation is clinicopathologically useful since it is predictive for relatively good chemotherapy response with comparably higher survival and a possible different microscopic appearance (22,23). Unfortunately, we did not have a chance to confirm the *BRCA* mutation further by PCR or by IHC in our cohort.

In breast cancer, the relationship between hormone expression profile and prognosis has been extensively studied, and classifications based on hormone receptor expression profile, are widely used in tumor management (24). Estrogen causes proliferation through cellular transforming molecules like the IL-6/STAT-3 signaling pathway (25). On the contrary, the progesterone-receptor induced by ER activation is known to induce apoptosis, and behaves as a tumor suppressor protein by activation through cellular caspase-8 (26). The protective effect of pregnancy on ovarian cancer is thought to be due to high levels of progesterone during pregnancy. Improved survival in ovarian cancer with positive estrogen or progesterone receptors has been reported in a few studies (27,28). In our study, the median OS of PR positive and negative cases were 66.8 months and 46.3 months respectively. ER expression did not confer a survival advantage such as PR, and the overall survival in ER-positive and negative cases were 44 and 37 months respectively. There was no relationship between PR expression and the morphological appearance of the tumor; however, it can be postulated that tumors expressing PR reflect a different high-grade serous carcinoma group and could benefit from endocrine treatment due to improved prognosis.

The prognostic value of ERCC1 expression in gynecological and non-gynecological cancers and its association with chemotherapy resistance have been investigated by various groups (29–32). Although studies are reporting that high ERCC protein expression predicts resistance to platinum-based therapies in various tumors, there is no general agreement on this issue (33–36). The relationship between ERCC1 IHC and survival in our study revealed a non-significant result, and the group with high ERCC1 expression was found to have 2 months shorter survival compared to the low ERCC1 expression group. In our patient population, there was no significant difference for TTP and TTFPFC among ERCC1 groups. Therefore, we could not demonstrate a predictive role of ERCC1 levels for platinum resistance in ovarian cancer.

The frequency of p53 mutation in high-grade serous carcinoma is about 99% and can be detected by immunohistochemistry as overexpression or complete absence that indicates gain-of-function or loss-of-function, respectively (37). In our study, 301 (99%) cases showed one of these two staining patterns consistent with the p53 mutation. Wild-type p53 was detected in only 4 cases. There was no significant difference in survival rates between patients with wild-type p53 staining and cases with aberrant staining. To summarize, wild-type p53 staining can be detected in high-grade serous carcinomas, although extremely rare, but a detailed morphological and immunohistochemical examination should be performed to exclude other epithelial tumors of the ovary in cases of focal staining.

In this study, the median OS of serous carcinoma with non-SET morphology was calculated as 44.7 months while the value for serous carcinoma with any SET morphology was calculated as 67 months. Moreover, the 21 months longer OS period in SET predominant tumors compared to SET non-dominant tumors suggests that high-grade serous carcinomas could be potentially diverse. There are 2 publications in the current literature discussing the relationship between the morphological appearance and the prognosis of high-grade serous carcinomas. In one of these, SET morphology and chemotherapy sensitivity were correlated. Howitt et al. primarily focused on SET, BRCA, and STIC relationships in a relatively small patient population (n=58) and reported a better clinical outcome for tumors with SET morphology in their cohort compared to classical morphology (9). Our results are similar with tumors having larger SET morphology areas having better TTP and TTFPFC in our study. In the second study, Ritterhause et al. assessed 104 patients’ survival outcomes. They showed a strong relationship with non-classic serous carcinoma histology and BRCA1 mutations but their groups of classic and SET morphologies did not show any significant difference for progression-free survival, platinum sensitivity, and OS (38).

In our study, higher ERCC1 was not associated with platinum resistance but higher SET morphology was associated with platinum sensitivity as reflected with a trend toward higher platinum-free survival. SET-predominant and the conventional serous morphology groups had 77.7 and 27.5 months of median TTFPFC, respectively (p=0.002). SET comes out as a new predictive factor for platinum sensitivity according to our findings. Moreover, PR positivity was protective from death only but SET predominance was the single independent predictive factor of protection in all of the survival endpoints.

A potential limitation of our study is that we were not able to test for the BRCA mutation or even other homologous recombination-deficiencies for corresponding tumor patterns. Additionally, due to the retrospective nature of the study, we were unable to elaborate in-situ lesions in ancient cases. However, the abundant number of cases and adequate follow-up periods may hinder these limitations.

Mounting evidence in recent reports supports that SET morphology has a genetic background different from classical serous morphology and lacks precursor lesions. A better understanding of the molecular pathogenesis of SET morphology will guide future efforts in diagnosis, biomarker-supported classification algorithms, and treatment strategies of high-grade serous ovarian carcinoma.

## Conflict of Interest

Study was funded by Hacettepe University Scientific Research Unit (THD-2015-8349). The authors declare no potential conflict of interest.
